# The Impact of Venoarterial and Venovenous Extracorporeal Membrane Oxygenation on Cerebral Metabolism in the Newborn Brain

**DOI:** 10.1371/journal.pone.0168578

**Published:** 2016-12-29

**Authors:** Aaron J. Reitman, Rachel Chapman, James E. Stein, Lisa Paquette, Ashok Panigrahy, Marvin D. Nelson, Philippe Friedlich, Jessica L. Wisnowski, Stefan Bluml

**Affiliations:** 1 Center for Fetal and Neonatal Medicine, Children’s Hospital Los Angeles, Los Angeles, California, United States of America; 2 Department of Pediatrics, Division of Neonatal Medicine, University of Southern California, Los Angeles, California, United States of America; 3 Department of Pediatrics, Division of Neonatal Medicine, Los Angeles County University of Southern California Medical Center, Los Angeles, California, United States of America; 4 Department of Surgery, Children’s Hospital Los Angeles, Los Angeles, California, United States of America; 5 Department of Surgery, University of Southern California, Los Angeles, California, United States of America; 6 Department of Radiology, Children’s Hospital Los Angeles, Los Angeles, California, United States of America; 7 Children’s Hospital of Pittsburgh of the University of Pittsburgh Medical Center, Pittsburgh, Pennsylvania, United States of America; 8 Rudi Schulte Research Institute, Santa Barbara, California, United States of America; University of Giessen Lung Center, GERMANY

## Abstract

**Background:**

Extracorporeal membrane oxygenation (ECMO) is an effective therapy for supporting infants with reversible cardiopulmonary failure. Still, survivors are at risk for long-term neurodevelopmental impairments, the cause of which is not fully understood.

**Objective:**

To elucidate the effects of ECMO on the newborn brain. We hypothesized that the cerebral metabolic profile of neonates who received ECMO would differ from neonates who did not receive ECMO. To address this, we used magnetic resonance spectroscopy (^1^H-MRS) to investigate the effects of venoarterial and venovenous ECMO on cerebral metabolism.

**Methods:**

41 neonates treated with ECMO were contrasted to 38 age-matched neonates.

**Results:**

All ^1^H-MRS data were acquired from standardized grey matter and white matter regions of interest using a short-echo (TE = 35 milliseconds), point-resolved spectroscopy sequence (PRESS) and quantitated using LCModel. Metabolite concentrations (mmol/kg) were compared across groups using multivariate analysis of covariance. Elevated creatine (*p* = 0.002) and choline (*p* = 0.005) concentrations were observed in the grey matter among neonates treated with ECMO relative to the reference group. Likewise, choline concentrations were elevated in the white matter (*p* = 0.003) while glutamate was reduced (*p* = 0.03). Contrasts between ECMO groups revealed lower osmolite concentrations (e.g. myoinositol) among the venovenous ECMO group.

**Conclusion:**

Neonates who underwent ECMO were found to have an abnormal cerebral metabolic profile, with the pattern of abnormalities suggestive of an underlying inflammatory process. Additionally, neonates who underwent venovenous ECMO had low cerebral osmolite concentrations as seen in vasogenic edema.

## Introduction

Neonatal extracorporeal membrane oxygenation (ECMO) is a critical and effective therapy for supporting infants with potentially reversible cardiopulmonary failure that is refractory to maximal medical therapy. It is most commonly used in the treatment of persistent pulmonary hypertension of the newborn (PPHN), meconium aspiration syndrome (MAS), and congenital diaphragmatic hernia (CDH), as well as some types of congenital heart disease[1]. Although the introduction of inhaled nitric oxide and high frequency ventilation has decreased the need for ECMO [2], more than 30,000 neonates have been treated with ECMO since the 1970s [3] and ECMO continues to be an important therapeutic modality in the in the neonatal intensive care unit. Data from the Extracorporeal Life Support Organization (ELSO) 2015 registry indicate that approximately 68% of neonates treated with ECMO survive to discharge/transfer [3]. Importantly, survivors are at risk for long-term neurodevelopmental impairments [4, 5], the cause of which is not fully understood.

ECMO involves the diversion of deoxygenated blood from the systemic circulation of the patient to the extracorporeal circuit followed by the return of oxygenated blood to the patient. For patients treated with venoarterial (VA) ECMO, dexogenated blood is drawn off the patient from the right internal jugular vein while oxygenated blood is returned via the right carotid artery, thus providing both cardiac and pulmonary support. In contrast, venovenous (VV) ECMO utilizes a double lumen catheter in the right internal jugular vein to draw off deoxygenated blood and to return oxgenated blood to the right atrium of the heart, thus providing only pulmonary support. Following results from a multisite clinical trial, VV ECMO has been the preferred modality for providing ECMO support in neonates who have adeqeuate cardiac function because it is associated with a similar rate of survival and fewer major neurologic complications [6, 7].

Still, long-term neurodevelopmental follow-up of newborns treated with VA and VV ECMO have demonstrated that both populations are at risk for academic difficulties, behavioral problems, and mental retardation [8] and it is not yet known whether these risks can be attributed to the same underlying mechanisms in both populations. Moreover, neonates supported with ECMO frequently have comorbid medical conditions, such as congenital heart disease and hypoxic-ischemic injury, which independently portend an increased risk for long-term neurodevelopmental disabilities [9, 10].

Magnetic resonance imaging (MRI) of the brain is used after ECMO to evaluate for intracranial lesions (e.g. stroke or hemorrhage) as these neonates receive anticoagulation during their ECMO therapy. Abnormal brain MRI findings for neonates who undergo ECMO have been associated with adverse neurological outcomes; however, neonates who do not have evidence of stroke or other intracranial findings remain at increased risk for adverse neurodevelopmental outcomes[11, 12]. With the advances in neuroimaging techniques, it is now possible to test for more subtle brain abnormalities in neonatal ECMO survivors, which may provide new insights into possible causes of long-term neurodevelopmental disabilities in this population.

Proton magnetic resonance spectroscopy (^1^H-MRS) is a non-invasive magnetic neuroimaging technique that provides biomarkers related to neuronal-axonal maturation, membrane synthesis, and energy metabolism [13]. ^1^H-MRS has been useful in characterizing cerebral abnormalities in the setting of neurological disorders [14], including, metabolic diseases in the newborn and hypoxic ischemic encephalopathy [15–17]. Our aim was to use ^1^H-MRS to elucidate the effects of VA and VV ECMO on the newborn brain. We hypothesized that the cerebral metabolic profile of neonates who received ECMO would differ from neonates who did not receive ECMO. Furthermore, we expected that neonates who underwent VA ECMO would demonstrate a more abnormal cerebral metabolic profile than neonates who underwent VV ECMO—owing to both their greater clinical severity and the fact that VA ECMO requires permanent ligation of the right carotid artery. Last, we explored whether cerebral metabolic profiles differed across patient groups (CDH, PPHN and MAS).

## Materials and Methods

### Patient Selection

**Fig 1:**
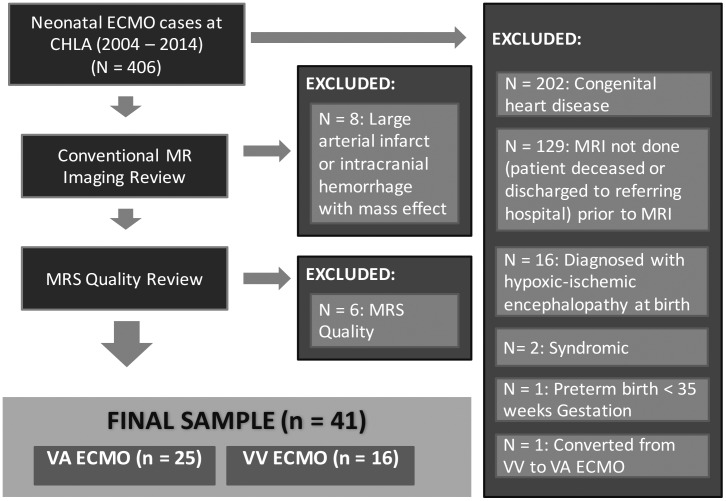
Patient selection. Depicted above is a schematic diagram demonstrating how the 41 neonates were selected from the total population of neonates who underwent ECMO at CHLA between 2004 and 2014.

A retrospective chart review was used to identify all neonates who underwent ECMO at Children’s Hospital Los Angeles (CHLA) between 2004 and 2014. To avoid confounding our findings, we excluded neonates with comorbid conditions that could independently, and with high probability, affect the developing brain, including preterm birth (≤ 35weeks gestation), congenital heart disease, hypoxic-ischemic encephalopathy and genetic syndromes. The remaining neonates were diagnosed with MAS, CDH or PPHN. (Of note, both the MAS and PPHN groups included infants with clinical sepsis). Next, we reviewed conventional MRI data to exclude neonates who had large arterial infarcts (i.e., involving of multiple segments of the anterior, middle or posterior cerebral arteries) or intracranial hemorrhages with mass effect, which could bias or distort the ^1^H-MRS data. Finally, one neonate was excluded because this patient required conversion from VV to VA ECMO. This resulted in a final sample of 41 neonates who were treated with either VA ECMO or VV ECMO (Fig 1).

We next selected a reference group of 38 neonates from the MR Spectroscopy Database at CHLA who were matched to the ECMO patients as close as possible with respect to gestational age at birth, age at MR scan and MRS field strength (1.5T versus 3T). Neonates in the reference group underwent MRI/^1^H-MRS for a variety of clinical indications (e.g., “apparent lift threatening event [ALTE]”), but were found to be free of cardiopulmonary disorders (including MAS, CDH, PPHN), neurological disorders and neurodevelopmental disabilities based on chart review, including extended follow-up when available, and many have been included in prior publications by our research group [18, 19].

### Ethics Statement

The Children’s Hospital Los Angeles (CHLA) Institutional Review Board approved the review of existing data, which had been acquired as part of the clinical work-up of hospital patients treated at CHLA between the years of 2004–2014. The requirement for consent was waived and all data were de-identified prior to analysis.

### MRI/MRS Acquisition

As standard clinical protocol at CHLA during this period (2004–2014), all neonates treated with ECMO were referred for MRI examination, which included conventional T1- and T2-weighted imaging, Diffusion Weighted Imaging (DWI, with Apparent Diffusion Coefficient [ADC] maps), and ^1^H-MRS, prior to discharge. MRI/^1^H-MRS examinations were conducted on either a 1.5T (Signa LX, GE Healthcare, Milwaukee, WI)) using a customized neonatal headcoil (Sree Medical Systems, Cleveland, OH) or a 3T MR System (Achieva, Philips Medical, Best, The Netherlands) using a standard 8-channel SENSE headcoil. ^1^H-MRS data were acquired from the medial parietal grey matter (GM) and parietal white matter (WM) using a single voxel point-resolved spectroscopy (PRESS) sequence with an echo time of 35 milliseconds, a repetition time of 1.5 seconds (2.0 seconds at 3T) and 128 signal averages, for a total acquisition time of 4–5 minutes, including scanner adjustments. Voxel size was consistent (~ 3 cm^3^) across field strength.

### MRI/MRS Data Processing

Conventional MRI data were reviewed by two authors (AP, JLW) who were blind to ECMO subgroup, and qualitatively scored by consensus for the presence of extra-axial fluid and ventricle size on a four-point scale (i.e., within normal limits, mildly increased, moderately increased, severely increased). Additionally, conventional MRI data were scored for the presence of intracranial blood products (extra-axial and intraventricular) and for parenchymal findings (e.g., punctate white matter lesions, hemorrhages, infarcts).

^1^H-MRS data were processed offline using LCModel software (Stephen Provencher Inc., Oakville, Ontario Canada). Briefly, LCModel is an automated, user-independent software that determines the concentrations (mmol/kg of tissue) of individual metabolites by fitting a library of concentration calibrated model spectra to the acquired ^1^H-MRS data [20]. Acquired data and LCModel fits were reviewed by two authors (JLW, SB) for artifacts and other errors in model fitting. Both authors were blinded to the mode of ECMO, neonate’s diagnosis, and outcome. Spectra of low quality were excluded by limiting the sample to spectra with a line width ≤ 5Hz and signal to noise ≥ 7. As per our published methods, 1.5T concentrations were corrected for water content[18, 19]; however, as we have subsequently shown that this adjustment has a negligible effect on metabolite concentrations for this small age range (see S2 Fig from [19]), this was not carried out for 3T data.

For this study, we focused our analyses on eight metabolites: creatine (corresponding to the total signal from phosphocreatine and free creatine), choline (corresponding to the total signal from glycerophosphocholine and phosphocholine), N-acetyl-aspartate (NAA), lactate, glutamate, glutamine, and myoinositol (corresponding to the total signal from myoinositol and glycine). These metabolites were selected *a priori*, because they are not only key markers of neuronal or astroglial function, but also can be reliably quantitated at both 1.5 and 3T, as demonstrated repeatedly by our group and others [17, 19, 21].

### ECMO Protocol

The decision to place a newborn on VA or VV ECMO was a clinical decision based on multiple considerations. In patients with respiratory failure without significant cardiac dysfunction, VV ECMO was considered the modality of choice except when patient’s habitus precludes insertion of a double lumen catheter. In contrast, in patients with need of cardiac support or when VV ECMO was not an option (due to patient size), VA ECMO was preferred.

The ECMO circuit included vascular access catheters, polyvinyl chloride tubing for effluent flow and reinfusion, a servo-regulated pump, heat exchanger and an oxygenator. From 2004 to early 2014, our institution used the Sorin S3 roller ECMO pump (Sorin Group USA, Incorporated, Arvada, CO) and in the remainder of 2014, we introduced the Sorin S5 centrifugal ECMO pump with Revolution head (Sorin Group USA, Incorporated, Arvada, CO). Most of the ECMO circuits employed from 2004 to 2010 used the silicone membrane Medtronic 0800 oxygenator (Medtronic, Inc., Minneapolis, MN,USA), while we have since used the QUADROX oxygenator (MAQUET Medical Systems USA, Wayne, NJ). All ECMO circuits used 0.25 inch tubing packs (Sorin Group USA, Incorporated, Arvada, CO or MAQUET Medical Systems USA, Wayne, NJ). The size of VA cannulae (Bio-Medicus, Medtronic, Inc., Minneapolis, MN,USA) or VV dual lumen cannulae (OriGen Dual Lumen Catheter, Origen Biomedical, Austin, TX) was chosen based on patient anatomy. The right common carotid artery was not repaired after VA ECMO therapy.

### Statistical Analyses

Clinical and conventional MRI findings were compared across ECMO groups using χ^2^, ANOVA or ANCOVA as appropriate. For the primary comparisons between ECMO patients and the reference cohort, metabolite concentrations were compared across groups using MANCOVA, controlling for age at MRI and MRI field strength. Post-hoc analyses (ANCOVA and Fisher’s Least Significant Difference) were used to compare differences pairwise across ECMO subtypes (VA vs VV) and diagnostic groups (MAS, CDH, PPHN). Statistical analyses were carried out in SPSS (v.23, IBM Corporation).

## Results

### Patient Characteristics

The patient characteristics for the ECMO patient groups and the reference group are presented in Table 1. Overall, the three groups were well-matched with regard to gestational age at birth (i.e., 39 weeks). However, there was a trend toward the VV ECMO patients being younger at the time of MRI measured as postconceptional age (GA at birth + postnatal age) or postnatal age alone (*p*’s < 0.1). For this reason, postconceptional age was included as a covariate in all subsequent analyses.

**Table 1:** Patient Characteristics.

	Reference (n = 38)	VA ECMO (*n* = 25)	VV ECMO (*n* = 16)	*p*-value
Sex	18 male/20 female	17 male/9 female	11 male/5 female	NS^a^
Gestational Age at Birth (weeks)	39.4 ± 1.5	38.9 ± 1.5	39.8 ± 1.3	NS^b^
Postconceptional Age at MRI (days)	309.4 ± 26.8	311.3 ± 25.3	294.3 ± 11.34	NS^b^
Birth Weight(grams)	n/a	3361 ± 528	3383 ± 389	NS^b^
Diagnoses	n/a	12 CDH (48%)	4 CDH (25%)	NS^a^
		7 PPHN (28%)	3 PPHN (19%)	
		6 MAS (24%)	9 MAS (56%)	
ECMO duration (hours)	n/a	294 ± 138	173 ± 82	**0.003**^b^
Serum sodium (at time of MRI)	n/a	137.4 ± 5.1	138.1 ± 2.3	NS^b^
Serum creatinine (at time of MRI)	n/a	0.26 ± 0.11	0.47 ± 0.16	**0.001**^c^
Serum creatinine (peak value prior to MRI)	n/a	0.68 ± 0.21	0.84 ± 0.11	**0.028**^c^

Values above represent mean ± standard deviation. n/a = not available. MAS = meconium aspiration syndrome, CDH = congenital diaphragmatic hernia, PPHN = persistent pulmonary hypertension of the newborn, NS = non significant.

^a^*p*-value derived from χ^2^

^b^*p*-value derived from ANOVA

^c^*p*-value derived from ANCOVA, controlling for age

Within the ECMO patient groups, on average, VA ECMO patients were on ECMO for a longer duration (*p* = 0.003), and there was a trend toward there being higher proportions of CDH and PPHN patients and a lower proportion of MAS patients in the VA ECMO group relative to the VV ECMO group (*p* < 0.1). Clinically, serum creatinine was mildly elevated among the VV ECMO patients in relation to both the VA ECMO group and normative values [22]: however, none of the patients received renal replacement therapy.

As shown in Table 2, conventional MRI demonstrated increased extra axial fluid, increased lateral ventricle volumes and a higher incidence of punctate white matter lesions among ECMO patients relative to the reference group. However, there were no differences in the incidence of these findings across VA and VV ECMO groups. Finally, ADC values corresponding to the WM region of interest for the MRS were not different across groups.

**Table 2:** Conventional MRI Findings.

	Reference group	VA ECMO	VV ECMO	*3-way comparison*^a^	*VA vs VV ECMO*^a^
Extra-axial Fluid					
WNL	24	4	2	*p* < 0.001	NS
Mild increase	12	10	9		
Mod increase	1	9	4		
Severe increase	0	2	1		
Extra-axial Blood					
None	29	17	14	NS	NS
Subdural hematoma	8	8	2		
Ventricle Size					
WNL	34	4	6	*p* < 0.001	NS
Mild increase	3	15	8		
Mod increase	0	6	2		
Severe increase	0	0	0		
Intraventricular Hemorrhage					
None	36	24	15	NS	NS
Choroid Plexus Hemorrhage	0	1	1		
Parenchymal Findings					
None	37	20	12	*p* < 0.05	NS
Punctate white matter lesions	0	4	3		
Hemorrhage (without mass effect)	0	1	0		
Laminar necrosis (cortex)	0	0	1		
MR Field Strength (3T: 1.5T)	21:16	14:11	10:6	NS	NS
White matter ADC ^b^ (mean ± SEM)	1.37 ± 0.04	1.37 ± 0.05	1.36 ± 0.06	NS	NS

WNL = within normal limits, ADC = apparent diffusion coefficient, NS = non significant

^a^ *p*-value derived from χ^2^, except for ADC, which was assessed using ANCOVA

^b^ADC values corrected for PCA at MRI and MRI field strength

### Cerebral Metabolic Profile Associated with ECMO

The cerebral metabolic profile of the neonatal ECMO group differed significantly from the reference group in both the GM (*p* = 0.001) and WM (*p* = 0.001) regions of interest. Follow-up analyses revealed elevated concentrations of creatine (GM) and choline (GM and WM) among the ECMO patients, while glutamate was reduced (WM) (Table 3; see also S1 and S2 Tables).

**Table 3:** Cerebral metabolite concentrations in neonates with ECMO and reference group.

		Creatine^a^	Choline^a^	NAA^a^	Lactate^a^	Glutamate^a^	Glutamine^a^	Myoinositol^a^
Grey Matter	Reference mmol/kg	4.75 (0.12)	1.82 (0.05)	4.01 (0.12)	0.50 (0.05)	5.59 (0.26)	3.44 (0.29)	9.51 (0.24)
	ECMO mmol/kg	5.29 (0.11)	2.00 (0.04)	3.99 (0.11)	0.46 (0.05)	5.08 (0.24)	3.29 (0.27)	9.42 (0.23)
	*p*-value^b^	**0.002**	**0.005**	0.92	0.57	0.16	0.72	0.78
White Matter	Reference mmol/kg	4.82 (0.11)	1.98 (0.05)	4.27 (0.10)	0.59 (0.06)	5.46 (0.22)	4.12 (0.23)	7.93 (0.20)
	ECMO mmol/kg	5.13 (0.11)	2.20 (0.05)	4.04 (0.10)	0.53 (0.06)	4.79 (0.22)	4.03 (0.22)	8.18 (0.20)
	*p*-value^b^	0.052	**0.003**	0.11	0.46	**0.03**	0.79	0.40

NAA = N-acetyl aspartate

^a^values represent adjusted means [controlling for age at MRI and MR field strength] ± (standard error of the mean).

^b^*p*-value derived from ANCOVA.

### The Effects of VA versus VV ECMO

**Fig 2:**
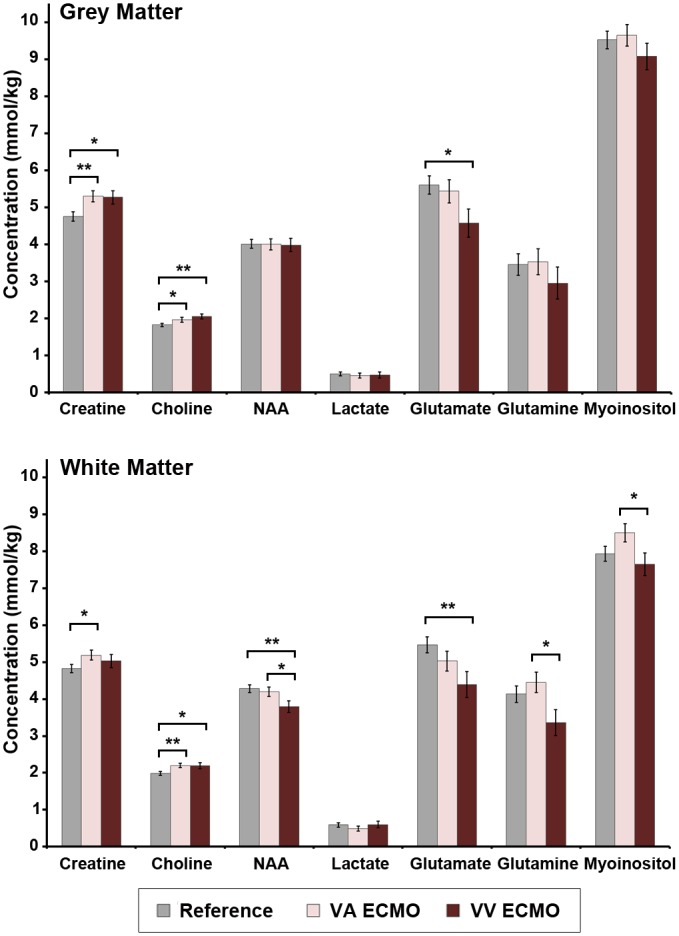
Cerebral metabolite concentrations in the grey matter and white matter among neonates treated with VA ECMO, VV ECMO and reference group. Values above represent adjusted means (controlling for age at MRI and MR field strength) ± standard error of the mean, stratified by group. *p*-values were derived from Fisher’s Least Significant Difference. Note: significant differences shown by an asterisk, all others non-significant, *p < 0.05; **p < 0.01.

Likewise, when the ECMO groups were considered separately, there were differences in the metabolic profiles of the VA and VV ECMO groups relative to the reference group (GM: *p* = 0.004; WM: *p* < 0.001). Post-hoc contrasts between VA ECMO patients and the reference group revealed higher concentrations of creatine (GM) and choline (WM) in the VA ECMO group (Fig 2). Likewise, post-hoc contrasts between VV ECMO patients revealed higher choline (GM & WM) and lower glutamate (GM & WM), glutamine (WM) and NAA (WM). Finally, post-hoc contrasts between ECMO groups revealed lower concentrations of myoinositol (WM), glutamine (WM) and NAA (WM) among the VV ECMO patients relative to the VA ECMO group.

### The Effects of Diagnostic Subgroups

**Fig 3:**
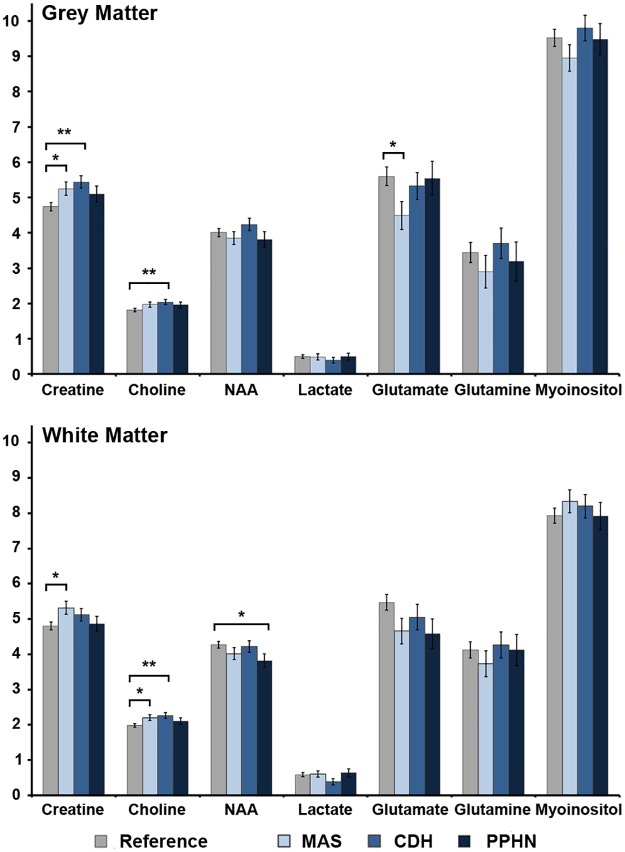
Cerebral metabolite concentrations in the grey matter and white matter among diagnostic groups as compared to the reference group. Values above represent adjusted means (controlling for age at MRI and MR field strength) ± standard error of the mean, stratified by group. *p*-values were derived from Fisher’s Least Significant Difference. MAS = meconium aspiration syndrome; CDH = congenital diaphragmatic hernia, PPHN = persistent pulmonary hypertension of the newborn.

Finally, we considered the effect of patient diagnosis. As expected the cerebral metabolic profile associated with MAS, CDH and PPHN differed from the reference group (GM: *p* = 0.04; WM: *p* = 0.01). Post-hoc analyses revealed higher creatine (GM & WM) and choline (WM) and lower glutamate (GM) concentrations among neonates diagnosed with MAS relative to the reference group (Fig 3). Likewise, there were higher concentrations of creatine (GM) and choline (WM) among the CDH ECMO group relative to the reference and lower concentrations of NAA (WM) in the PPHN ECMO group relative to the reference group. There were no significant pairwise differences among ECMO-patient groups.

## Discussion

The primary aim of this study was to investigate the effect of ECMO on newborn brain metabolism. Using clinically available ^1^H-MRS, we observed differences in key cerebral metabolites among neonates treated with ECMO as compared to our reference group. Specifically, neonates who had been treated with ECMO were found to have increased creatine and choline in the grey matter relative to our reference group. Likewise, there was higher choline in the white matter and reduced glutamate. Although these data only represent a short-term outcome after the completion of ECMO therapy, they add to a growing body of literature detailing the neurological complications of ECMO.

Longitudinal studies have revealed higher rates of neurodevelopmental disabilities as well as more subtle impairments in behavior and executive functioning among school-aged survivors of ECMO [4, 5]. Considering that the rates of intracranial hemorrhage and ischemic infarcts have remained low (7.4% and 5.7%), it is unlikely that acute, clinically-apparent neurologic complications account for all of the later neurodevelopmental impairments [23]. Our findings suggest that there may be subtle differences in the brains of neonates who underwent ECMO, even in the absence of large intracranial hemorrhages or infarcts, which could set the stage for later neurodevelopmental impairments.

In ^1^H-MRS, choline is considered a membrane marker because it incorporates the precursors or degradation products of membrane phospholipids. Choline concentrations decline during infancy, coincident with neuronal/axonal maturation and myelination [18, 19, 24]. Additionally, prior studies have shown that choline concentrations are elevated in the setting of CNS inflammation, with the magnitude of the choline signal related to glial proliferation [25] and demyelination/remyelination [26, 27]. By contrast, creatine, an energy metabolite, does not originate in the brain but instead is continuously synthesized in the kidney and liver, transported into the brain and finally, excreted from the body as creatinine [28]. We observed higher creatine and choline in the grey matter and higher choline in the white matter of neonates who were treated with ECMO. Although the pathophysiological basis for these observations cannot be determined from the present study, the findings suggest two potential hypotheses. First, considering that neonates with ECMO may have acute kidney injury [29] and reduced creatinine clearance, it is possible that the elevation in cerebral creatine reflects impairment in creatine-creatinine clearance. We recorded serum creatinine levels on the day of the MRI and peak serum values during ECMO therapy. While the VV ECMO patients had higher serum creatinine levels (Table 1), post-hoc analyses demonstrated no direct correlation between serum creatinine and either GM or WM creatine (data not shown). On the other hand, systemic inflammation (e.g., elevated c-reactive protein, elevated pro-inflammatory cytokines) has been documented in neonates with ECMO [30, 31], as well as in neonates with clinical sepsis [32] and studies have demonstrated that creatine is often elevated alongside choline in the setting of cerebral inflammation [25, 27]. Taken together, the elevations in creatine and choline suggest that the systemic inflammatory response could extend to the central nervous system, as demonstrated in animal models [33].

At the outset of this project, one of our underlying hypotheses was that VA ECMO would convey a higher risk of neurologic abnormalities than VV ECMO. There are several disadvantages in the use of VA ECMO therapy [34] primarily, the need for right carotid arterial ligation. Early investigations of neonatal ECMO noted an increase in infarcts, hemorrhages, and seizures to the right hemisphere in infants who underwent ligation of the right carotid artery [35]; however, a recent study suggests otherwise [36]. Moreover, despite ligation of the right carotid artery, Roelants-van Rijn and colleagues [37] found no differences in cerebral metabolism between the right and left basal ganglia amongst a small sample of neonates who were treated with VA ECMO. Our study did not contrast cerebral metabolism in the right and left hemisphere. However, we did find differences between infants in the VA ECMO and the reference group, namely, an increase in creatine in the grey matter and an increase in choline in the white matter.

**Fig 4:**
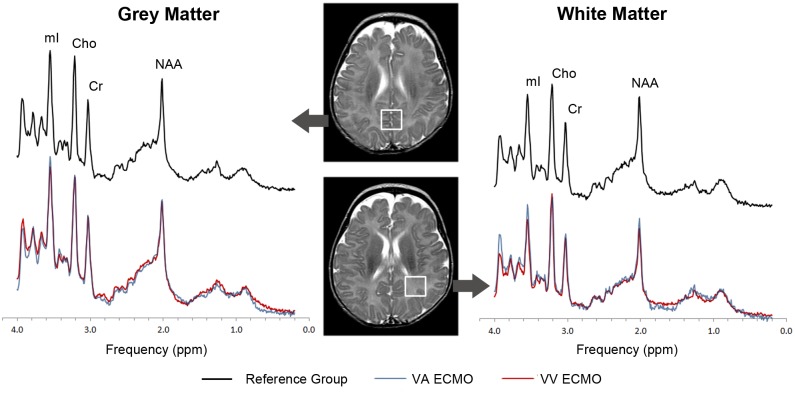
Typical ^1^H-MRS Spectra among neonates with VA ECMO, VV ECMO and the reference group. Pictured above are averaged spectra (matched for post-menstrual age), obtained from the grey and white matter regions of interest (see inlay). Note that, in the white matter, myoinositol, and NAA are reduced in the VV group relative to the VA group, consistent with a hypo-osmolar state. NAA = n-acetyl-aspartate, Cho = choline, Cr = creatine, mI = myoinositol.

More surprising, we observed multiple abnormalities in cerebral metabolism when we directly compared the VV ECMO group to the VA ECMO group as well as in the VV ECMO group relative to the reference group (Fig 4). In VV ECMO, oxygenated blood is returned to the right atrium instead of to the proximal right common carotid artery as with VA ECMO. This results in an increase in right atrial pressure and consequently increased pressure in the superior vena cava and sagittal sinus, i.e., cerebral venous congestion [38]. Neuroimaging has shown neonates on VV ECMO compared to VA ECMO have an increase in their subarachnoid space and widening of their inter-hemispheric fissures [38, 39] which is presumed to be secondary to cerebral venous congestion. Although we did not observe significant differences between VA and VV ECMO with regard to extraaxial fluid or DWI-ADC measurements, we did observe lower myoinositol, glutamine and NAA in the white matter of our VV ECMO patients relative to VA ECMO patients as well as a decrease in NAA and glutamate among VV ECMO patients relative to the reference group. Myoinositol, glutamate, glutamine and NAA can all function as osmolites, and studies have demonstrated that the brain compensates for osmotic stress by losing these solutes, thereby mitigating cerebral edema [40]. Moreover, recovery of the organic osmolites following osmotic stress, occurs very slowly over a period of many days and thus distrubances in osmolite concentrations typically persist beyond the period of acute osmotic stress [41]. Further research is needed to establish if and when osmolite concentrations normalize following ECMO.

Neonates who receive VA compared to VV ECMO are typically more ill [42] as they require both cardiac and pulmonary support. Our VA ECMO patients were on ECMO on average 120 hours longer than the VV ECMO group, and thus not only required greater cardiopulmonary support but also for a longer duration. Thus, in this context, it was striking that the neonates who underwent VV ECMO displayed greater abnormalities in cerebral metabolism then those who underwent VA ECMO. Further research is needed to determine whether this metabolic derangement represents a reversible short-term outcome or whether it persists during the course of postnatal development.

Finally, we examined patient-factors, namely, differences between neonates with MAS, CDH and PPHN. Longitudinal neurodevelopment in neonates with CDH has been well-studied, and both short-term and long-term outcome studies suggest that this population, in particular, has the highest risk for neurodevelopment impairment [43]. Childhood survivors of CDH who are treated with ECMO have higher rates of neurodevelopmental impairments as well as more subtle difficulties in concentration and behavior compared to age-matched typically developing children [43]. In contrast, less is known regarding long-term outcomes in children with MAS treated with ECMO. We observed increases in creatine and choline in neonates with MAS and CDH relative to our reference group. Laboratory research has shown that inflammation may play a key role in the pathogenesis of MAS[44]. Additionally, several recent studies have suggested a role for inflammation in the pathogenesis of PPHN in the setting of CDH, starting in the intrauterine environment [45, 46]. While not definitive, our results suggest that the systemic inflammatory response extends into the central nervous system. Further research is warranted.

### Limitations

Our ^1^H-MRS data were acquired on 1.5T and 3T MR platforms, which could affect calculated metabolite concentrations. To account for this possibility, field strength was included as a covariate in all statistical analyses. Furthermore, as shown in S1 and S2 Tables, the trends for each metabolite were consistent across the dataset, i.e., a lower value at 1.5T was confirmed by a lower value in 3T. Another limitation of the current data set includes the relative small sample size in the ECMO patient group, which is due to our strict inclusion criteria and the fact that many of our patients were transferred back to their referring institution before undergoing MR imaging. It should also be noted that infants with a history of MAS, CDH, and PPHN were excluded from the reference group. Given this, we cannot determine to what extent the observed metabolic differences are attributable to ECMO therapy or to the underlying disease process, i.e., MAS, CDH, or PPHN, which necessitated ECMO. Future studies are needed to compare neonates with MAS, CDH and PPHN who are treated with ECMO to those who are not. Additionally, to better match the reference group to our ECMO patients with regard to GA at birth, postconceptional age at MRI and MR field strength, we did include neonates who were referred for MRI following ALTE. Although to our knowledge, the subsequent work-up was negative, we cannot rule out the possibility that they suffered transient hypoxemia, which in turn, may have reduced our ability to detect differences in the ECMO patients relative to the reference group. Future studies are needed to prospectively compare neonates who undergo ECMO healthy, term-born neonates. Finally, long-term follow-up is ongoing. Once completed, these data will help clarify the relations between the observed cerebral abnormalities and long-term neurodevelopmental outcomes.

## Conclusion

As survival rates for neonates treated with ECMO continue to improve, increased attention is being placed on long-term neurodevelopmental outcomes. This is, to the best of our knowledge, the first study demonstrating abnormalities in the cerebral metabolic profile of neonates who received ECMO therapy as compared to a reference group of term neonates. Interestingly, we demonstrated marked differences between VA and VV ECMO with the metabolic pattern strongly supporting the theory of increased venous congestion in VV ECMO. Finally, we observed metabolic abnormalities among neonates with CDH and MAS, which may be reflective of an underlying cerebral inflammatory process. Overall, these findings suggest that further research is needed to understand the impact of ECMO on the developing brain and highlight the importance of using advanced neuroimaging techniques like MRS in the evaluation of neonates who were treated with ECMO.

## Supporting Information

S1 Table Cerebral metabolite concentrations in grey matter ROI in neonates with ECMO and reference group. Note: values above represent adj. mean ± SEM, with values in full model adjusted for postconceptional age at MRI and MR field strength, while values in reduced model are only adjusted for postconceptional age at MRI. ROI = Region of Interest;PCr = Phosphocreatine; Cr = creatine; GPC = glycerophosphocholine; PC = phosphocholine; NAA = n-acetylaspartate; mI = myoinositol;ECMO = extracorporeal membrane oxygenation.
(DOCX)

S2 Table Cerebral metabolite concentrations in white matter ROI in neonates with ECMO and reference group. Note: values above represent adj. mean ± SEM, with values in full model adjusted for postconceptional age at MRI and MR field strength, while values in reduced model are only adjusted for postconceptional age at MRI. ROI = Region of Interest PCr = Phosphocreatine; Cr = creatine; GPC = glycerophosphocholine; PC = phosphocholine; NAA = n-acetylaspartate; mI = myoinositol; ECMO = extracorporeal membrane oxygenation.
(DOCX)
