# Supplementary material for: The Impact of Venoarterial and Venovenous Extracorporeal Membrane Oxygenation on Cerebral Metabolism in the Newborn Brain
Source: PLoS One. 2016 Dec 29;11(12):e0168578. doi: 10.1371/journal.pone.0168578 (PMC5199081; doi:10.1371/journal.pone.0168578)
Supplement: S1 Table — Note: values above represent adj. mean ± SEM, with values in full model adjusted for postconceptional age at MRI and MR field strength, while values in reduced model are only adjusted for postconceptional age at MRI. ROI = Region of Interest;PCr = Phosphocreatine; Cr = creatine; GPC = glycerophosphocholine; PC = phosphocholine; NAA = n-acetylaspartate; mI = myoinositol;ECMO = extracorporeal membrane oxygenation. (DOCX) [file pone.0168578.s001.docx]

| **Table S1. Cerebral metabolite concentrations in grey matter ROI in neonates with ECMO and reference group** | | | | | | | | | | |  | |
| --- | --- | --- | --- | --- | --- | --- | --- | --- | --- | --- | --- | --- |
| **Full Model** | **Creatine (PCr + Cr)** | **Choline (GPC + PC)** | | **NAA** | | **Lactate** | **Glutamate** | | **Glutamine** | **Myoinositol (mI + Glycine)** | |  |
| Reference | 4.75 ± 0.12 | 1.82 ± 0.05 | | 4.01 ± 0.12 | | 0.50 ± 0.05 | 5.59 ± 0.26 | | 3.44 ± 0.29 | 9.51 ± 0.24 | |  |
| ECMO | 5.29 ± 0.11 | 2.00 ± 0.04 | | 3.99 ± 0.11 | | 0.46 ± 0.05 | 5.08 ± 0.24 | | 3.29 ± 0.27 | 9.42 ± 0.23 | |  |
| ***p*-MR Field strength** | **0.001** | 0.159 | | **0.0001** | | **0.0001** | **0.008** | | 0.986 | **0.0001** | |  |
| ***p*-ECMO** | **0.002** | **0.005** | | 0.924 | | 0.571 | 0.162 | | 0.724 | 0.784 | |  |
|  |  |  | |  | |  |  | |  |  | |  |
| **Reduced model, 1.5T data only** | | | | |  | | | | | | |  |
| Reference | 4.85 ± 0.18 | 1.84 ± 0.07 | | 4.42 ± 0.18 | | 0.22 ± 0.08 | 5.07 ± 0.41 | | 3.30 ± 0.50 | 10.66 ± 0.36 | |  |
| ECMO | 5.64 ± 0.16 | 2.05 ± 0.06 | | 4.56 ± 0.16 | | 0.30 ± 0.07 | 4.67 ± 0.37 | | 3.40 ± 0.45 | 11.27 ± 0.32 | |  |
| ***p*-ECMO** | **0.002** | **0.032** | | 0.573 | | 0.454 | 0.484 | | 0.894 | 0.214 | |  |
|  |  |  | |  | |  |  | |  |  | |  |
| **Reduced model, 3.0T data only** | | |  | | | | |  | | | |  |
| Reference | 4.58 ± 0.12 | 1.78 ± 0.06 | | 3.45 ± 0.12 | | 0.84 ± 0.06 | 6.18 ± 0.18 | | 3.56 ± 0.22 | 8.00 ± 0.27 | |  |
| ECMO | 4.85 ± 0.12 | 1.93 ± 0.06 | | 3.30 ± 0.12 | | 0.66 ± 0.06 | 5.67 ± 0.18 | | 3.17 ± 0.22 | 7.02 ± 0.27 | |  |
| ***p*-ECMO** | 0.139 | 0.08 | | 0.379 | | **0.037** | 0.057 | | 0.227 | **0.017** | |  |

**________________________________________________________________________________________________________________________________**

**Note: values above represent adj. mean ± SEM, with values in full model adjusted for postconceptional age at MRI and MR field strength, while values in reduced model are only adjusted for postconceptional age at MRI ROI=Region of Interest; PCr = Phosphocreatine; Cr = creatine; GPC = glycerophosphocholine; PC = phosphocholine; NAA = n-acetylaspartate; mI = myoinositol; ECMO = extracorporeal membrane oxygenation**
