# Supplementary material for: The Impact of Venoarterial and Venovenous Extracorporeal Membrane Oxygenation on Cerebral Metabolism in the Newborn Brain
Source: PLoS One. 2016 Dec 29;11(12):e0168578. doi: 10.1371/journal.pone.0168578 (PMC5199081; doi:10.1371/journal.pone.0168578)
Supplement: S2 Table — Note: values above represent adj. mean ± SEM, with values in full model adjusted for postconceptional age at MRI and MR field strength, while values in reduced model are only adjusted for postconceptional age at MRI. ROI = Region of Interest PCr = Phosphocreatine; Cr = creatine; GPC = glycerophosphocholine; PC = phosphocholine; NAA = n-acetylaspartate; mI = myoinositol; ECMO = extracorporeal membrane oxygenation. (DOCX) [file pone.0168578.s002.docx]

**Table S2. Cerebral metabolite concentrations in white matter ROI in neonates with ECMO and reference group**

| **Full Model** | **Creatine (PCr + Cr)** | **Choline (GPC + PC)** | **NAA** | | **Lactate** | **Glutamate** | **Glutamine** | **Myoinositol (mI + Glycine)** |
| --- | --- | --- | --- | --- | --- | --- | --- | --- |
| Reference | 4.82 ± 0.11 | 1.98 ± 0.05 | 4.27 ± 0.10 | | 0.59 ± 0.06 | 5.46 ± 0.22 | 4.12 ± 0.23 | 7.93 ± 0.20 |
| ECMO | 5.13 ± 0.11 | 2.20 ± 0.05 | 4.04 ± 0.10 | | 0.53 ± 0.06 | 4.79 ± 0.22 | 4.03 ± 0.22 | 8.18 ± 0.20 |
| ***p*-MR Field Strength** | **0.0001** | **0.001** | **0.0001** | | **0.0001** | 0.701 | 0.363 | **0.0001** |
| ***p*-ECMO** | 0.052 | **0.003** | 0.11 | | 0.459 | **0.033** | 0.785 | 0.398 |
|  |  |  |  | |  |  |  |  |
| **Reduced model, 1.5T data only** | | | |  | | | | |
| Reference | 5.06 ± 0.19 | 2.09 ± 0.08 | 4.86 ± 0.15 | | 0.39 ± 0.09 | 5.44 ± 0.35 | 4.09 ± 0.37 | 8.93 ± 0.33 |
| ECMO | 5.37 ± 0.17 | 2.29 ± 0.07 | 4.56 ± 0.14 | | 0.26 ± 0.08 | 4.53 ± 0.32 | 4.26 ± 0.35 | 9.47 ± 0.31 |
| ***p*-ECMO** | 0.234 | 0.068 | 0.167 | | 0.316 | 0.065 | 0.747 | 0.239 |
|  |  |  |  | |  |  |  |  |
| **Reduced model, 3.0T data only** | | | | | | | | |
| Reference | 4.46 ± 0.10 | 1.83 ± 0.05 | 3.42 ± 0.09 | | 0.87 ± 0.07 | 5.45 ± 0.19 | 4.10 ± 0.22 | 6.56 ± 0.17 |
| ECMO | 4.81 ± 0.10 | 2.05 ± 0.05 | 3.37 ± 0.09 | | 0.94 ± 0.07 | 5.23 ± 0.20 | 3.73 ± 0.23 | 6.33 ± 0.18 |
| ***p*-ECMO** | **0.017** | **0.006** | 0.702 | | 0.724 | 0.438 | 0.26 | 0.379 |

**Note: values above represent adj. mean ± SEM, with values in full model adjusted for postconceptional age at MRI and MR field strength, while values in reduced model are only adjusted for postconceptional age at MRI ROI=Region of Interest; PCr = Phosphocreatine; Cr = creatine; GPC = glycerophosphocholine; PC = phosphocholine; NAA = n-acetylaspartate; mI = myoinositol; ECMO = extracorporeal membrane oxygenation**
